# MAGNIMS consensus recommendations on the use of brain and spinal cord atrophy measures in clinical practice

**DOI:** 10.1038/s41582-020-0314-x

**Published:** 2020-02-24

**Authors:** Jaume Sastre-Garriga, Deborah Pareto, Marco Battaglini, Maria A. Rocca, Olga Ciccarelli, Christian Enzinger, Jens Wuerfel, Maria P. Sormani, Frederik Barkhof, Tarek A. Yousry, Nicola De Stefano, Mar Tintoré, Massimo Filippi, Claudio Gasperini, Ludwig Kappos, Jordi Río, Jette Frederiksen, Jackie Palace, Hugo Vrenken, Xavier Montalban, Àlex Rovira, on behalf of the MAGNIMS study group

**Affiliations:** 1 Multiple Sclerosis Centre of Catalonia (Cemcat), Department of Neurology/Neuroimmunology, Hospital Universitari Vall d’Hebron, Universitat Autònoma de Barcelona, Barcelona, Spain; 2 Section of Neuroradiology and Magnetic Resonance Unit, Department of Radiology, Hospital Universitari Vall d’Hebron, Universitat Autònoma de Barcelona, Barcelona, Spain; 3 Department of Medicine, Surgery and Neuroscience, University of Siena, Siena, Italy; 4 Neuroimaging Research Unit, Institute of Experimental Neurology, Division of Neuroscience, IRCCS San Raffaele Scientific Institute, Milan, Italy; 5 NMR Research Unit, University College London Queen Square Institute of Neurology, London, UK; 6 National Institute for Health Research Biomedical Research Centre, University College London Hospitals, London, UK; 7 Department of Neurology and Division of Neuroradiology, Vascular and Interventional Radiology, Department of Radiology, Medical University of Graz, Graz, Austria; 8 Medical Image Analysis Center (MIAC AG) and Department of Biomedical Engineering, University of Basel, Basel, Switzerland; 9 Biostatistics Unit, Department of Health Sciences, University of Genoa, Genoa, Italy; 10 IRCCS, Ospedale Policlinico San Martino, Genoa, Italy; 11 Amsterdam Neuroscience, MS Center Amsterdam, Department of Radiology and Nuclear Medicine, Amsterdam UMC, Amsterdam, Netherlands; 12 Institutes of Neurology and Healthcare Engineering, University College London, London, UK; 13 Lysholm Department of Neuroradiology, University College London Hospitals National Hospital for Neurology and Neurosurgery, University College London Institute of Neurology, London, UK; 14 Vita-Salute San Raffaele University, Milan, Italy; 15 Multiple Sclerosis Center, Department of Neurosciences, San Camillo-Forlanini Hospital, Rome, Italy; 16 Neurologic Clinic and Policlinic, Departments of Medicine, Clinical Research and Biomedical Engineering, University Hospital, University of Basel, Basel, Switzerland; 17 Department of Neurology, Rigshospitalet-Glostrup and University of Copenhagen, Glostrup, Denmark; 18 Nuffield Department of Clinical Neurosciences, University of Oxford, Oxford, UK; 19 Division of Neurology, St Michael’s Hospital, University of Toronto, Toronto, Canada

**Keywords:** Multiple sclerosis, Brain imaging, Biomarkers

## Abstract

Early evaluation of treatment response and prediction of disease evolution are key issues in the management of people with multiple sclerosis (MS). In the past 20 years, MRI has become the most useful paraclinical tool in both situations and is used clinically to assess the inflammatory component of the disease, particularly the presence and evolution of focal lesions — the pathological hallmark of MS. However, diffuse neurodegenerative processes that are at least partly independent of inflammatory mechanisms can develop early in people with MS and are closely related to disability. The effects of these neurodegenerative processes at a macroscopic level can be quantified by estimation of brain and spinal cord atrophy with MRI. MRI measurements of atrophy in MS have also been proposed as a complementary approach to lesion assessment to facilitate the prediction of clinical outcomes and to assess treatment responses. In this Consensus statement, the Magnetic Resonance Imaging in MS (MAGNIMS) study group critically review the application of brain and spinal cord atrophy in clinical practice in the management of MS, considering the role of atrophy measures in prognosis and treatment monitoring and the barriers to clinical use of these measures. On the basis of this review, the group makes consensus statements and recommendations for future research.

## Abstract (web summary)

In this Consensus statement, the Magnetic Resonance Imaging in MS (MAGNIMS) study group reviews the application of brain and spinal cord atrophy in clinical practice in the management of MS and makes consensus statements and recommendations for future research.

## Introduction

The inflammatory component of multiple sclerosis (MS) pathology can be focal or diffuse and is associated with neurodegenerative processes that ultimately lead to irreversible tissue damage and neuronal loss^1^. Neurodegeneration was originally thought to be a late-stage phenomenon with limited clinical relevance, but it is now recognized as being associated with acute inflammation from the early stages of MS and as the main driver of irreversible disability^2–5^. In parallel with improvements in our understanding of the mechanisms of neurodegeneration, advances in imaging techniques have enabled in vivo assessment of brain and spinal cord area and volumes using MRI. Although brain and spinal cord volume loss observed with MRI cannot be equated with atrophy^6^, because the latter implies pathologically proven and irreversible tissue loss, changes in these MRI measures are associated with atrophy^7^ and the level of disability in MS^8,9^.

MRI-based quantification of inflammatory activity in MS — on the basis of lesion counts and lesion volumes — is established as the main efficacy outcome in phase II clinical trials^10^. Currently, brain and spinal cord volume measures have no role in the MS diagnostic criteria^11,12^ or disease course classification^13^, but a body of evidence that these measures are valuable for early evaluation of treatment responses and prediction of disease evolution has been steadily growing alongside improvements in methodology that could facilitate widespread implementation of these measures in clinical practice^14,15^. A key difficulty arises in this implementation because translation of group-based results into actionable, patient-level information must be made with extreme caution.

In this Consensus Statement, we, on behalf of the Magnetic Resonance in Imaging in MS (MAGNIMS) study group, provide specific recommendations for the implementation of brain and spinal cord atrophy measures in the clinical management of patients with MS and on the directions of future research to improve our knowledge in this field. The recommendations are based on a critical review of the literature and the personal experience of MAGNIMS study group members. We discuss the difficulties of translating group-based data into clinical application and highlight where particular caution is appropriate. We first discuss the role of atrophy measures on prognosis, then treatment monitoring and, finally, the barriers to implementation in clinical practice. Each of these three sections comprises a review of the available evidence and a set of consensus guidelines.

## Methods

A multicentre international panel on the implementation of brain and spinal cord atrophy measures in clinical practice convened in Barcelona, Spain, under the auspices of MAGNIMS, an independent European network of clinical research groups with a common interest in the study of MS with MRI. The panel was made up of experts in the diagnosis and management of MS, including neuroradiologists, neurologists, physicists, imaging methodologists and statisticians, who were selected by the workshop organizers (with approval from all members of the Steering Committee) on the basis of their personal expertise, from MAGNIMS centres from seven different countries. The purpose of this face-to-face meeting was to review and discuss all published data on brain and spinal cord atrophy in MS and to consider whether the previously published recommendations^16,17^ on its use for diagnosis, prognosis and monitoring of patients with MS needed to be revised and updated in view of technical advances and numerous clinical studies of atrophy in MS. The panel agreed that updated recommendations were necessary. After this meeting, the panel members formulated specific recommendations in relation to the implementation of brain and spinal cord atrophy measures in clinical practice.

The authors of the Consensus statement are members of the MAGNIMS Study Group. The network is independent of any other organization and, at the time of the workshop mentioned above, was run by a Steering Committee whose members were À. Rovira (Barcelona, co-chair), C. Enzinger (Graz, co-chair), F. Barkhof (Amsterdam), O. Ciccarelli (London), N. de Stefano (Siena), M. Filippi (Milan), J. Frederiksen (Copenhagen), C. Gasperini (Rome), L. Kappos (Basel), J. Palace (Oxford), M.A. Rocca (Milan), J. Sastre-Garriga (Barcelona), H. Vrenken (Amsterdam) and T. Yousry (London). The first draft of the recommendations was written by the principal authors (J.S.-G., D.P. and M.B.) on the basis of the panellists’ presentations and contributions to discussions on specific topics, which were assigned to individuals according to each member’s area of expertise. The initial draft was then circulated among all authors (who were all presenters and/or discussants at the meeting). Modifications were made iteratively until consensus was reached on all recommendations; all panel members agreed on the full contents of the final recommendations.

## Defining and predicting MS severity

### Evidence review

#### Global brain volume measures to define and predict MS severity

The initial studies to investigate clinical correlates of brain atrophy in MS focused on patients with well-established disease and severe clinical manifestations, particularly in the cognitive sphere^18–20^, but later studies included disability, as measured with the Expanded Disability Status Scale^8^. Evidence from these studies made it clear that neurodegenerative processes occur in the earliest phases of MS^21^, even before the disease becomes symptomatic^22^.

**Fig. 1:**
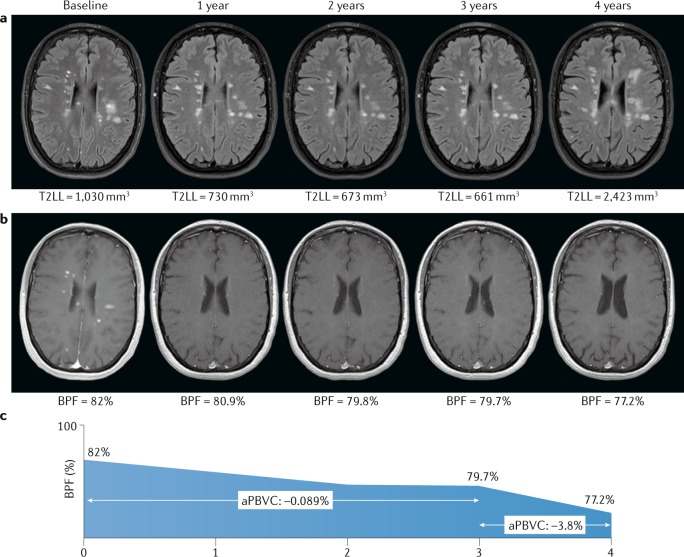
Lesion load and brain atrophy in relapsing–remitting multiple sclerosis. **a** | Transverse T2-weighted fluid attenuation inversion recovery images from a patient with highly active relapsing–remitting multiple sclerosis (MS) who started a disease-modifying therapy at baseline. The T2 lesion load (T2LL) is stable during the first 3 years of treatment while the patient remained clinically stable (no relapses and no disability worsening), but markedly increases at the fourth year after treatment discontinuation associated with clinical activity (the rebound effect). **b** | Contrast-enhanced T1-weighted images from the same patient showing the change in brain parenchymal fraction (BPF) over time. The decrease in global brain volume in the first 3 years is mild (annualized percentage of brain volume change (aPBVC) −0.089%), but the volume loss at the fourth year is severe (aPBVC −3.8%), matching the change in T2LL and clinical evolution. The severe loss observed in year 4 is well beyond the −0.4% suggested as a pathological cut-off for brain volume loss in MS^24^. **c** | Graphical representation of the changes in BPF over time, emphasizing the dramatic loss of volume in year 4.

Yearly global brain volume loss in healthy ageing individuals ranges from –0.05% at 20–30 years of age to –0.3% at 60–70 years of age^23^. A change of –0.4% per year has been proposed as the cut-off for pathological brain atrophy in MS^24^ (Fig. 1), although care must be taken before applying this threshold as a marker of therapeutic efficacy owing to the phenomenon of pseudoatrophy (see Brain volume as an outcome measure in randomized clinical trials)^25,26^. Multiple studies have shown that short-term changes (over as little as 1 year) in brain volume are predictive of clinical status (diagnosis of MS or disability status) at various follow-up times in clinically isolated syndromes^27,28^, relapsing–remitting MS (RRMS)^29^ and primary progressive MS^30–32^, either in isolation or together with lesion-related parameters^33,34^.

The findings above are group-based results, and translation of these findings to the individual level is not straightforward. In a study published in 2017, Sormani et al.^35^ made the first attempt to define individual cut-off values for brain volume changes according to patients’ baseline characteristics. Pooled baseline data from the placebo arms of two large international clinical trials that involved a total of 2,342 patients with RRMS showed that expected normalized brain volumes can be calculated from demographic (age and sex), clinical (Expanded Disability Status Scale score and disease duration) and neuroradiological (T2-weighted lesion volume) parameters for individuals. Deviation of the true brain volume from this expected value enabled classification of individuals with MS as having low, medium or high brain volume. Patients with low brain volume had a 2.4-fold higher risk of disability progression over the next 2 years than patients with high brain volume.

#### Spinal cord atrophy measures to define and predict MS severity

Early, seminal studies of cervical cord atrophy in MS already suggested that cervical spinal cord area is an important marker of disability status in MS^9^. Further studies demonstrated that spinal cord area and volume are affected differently in different MS subtypes, with the most profound atrophy in cross-sectional studies being seen in patients with progressive MS^36–39^. Since 2015, an association between reduced cervical cord area and increased disability and motor dysfunction, independent of brain atrophy, has been confirmed^40–43^. An association between cord atrophy and reduced peripapillary retinal nerve fibre layer thickness has been identified, indicating that cervical cord atrophy reflects, at least in part, global pathological processes and not only specific damage of long tracts^41^. Most studies of spinal cord area have focused on global cervical cord area measurements, but some work has highlighted that damage in particular locations in the spinal cord, such as cervical grey matter^44^, the thoracolumbar segment^45^ and the posterior and lateral cord segments^46^, are also relevant to disability.

Longitudinal studies indicate that atrophy rates in the spinal cord are higher than those in the brain and higher in progressive MS than in established RRMS^47,48^. Higher rates of cervical cord area loss have been associated with disability progression, independent of other clinical and MRI parameters^30,47^ including spinal cord lesions^49^. However, as for brain atrophy, use of such group-level evidence to inform clinical decisions at the individual level is not easy. Results that can be used at the individual level are slowly emerging; for example, Tsagkas et al.^43^ have shown that a 1% increase in the annual rate of spinal cord atrophy increases the risk of disability progression by 28%, reinforcing the notion that spinal cord atrophy is a reliable and independent tool for monitoring disease progression.

#### Regional and tissue-specific brain volumetry measures to define and predict MS severity

Early cross-sectional studies of brain white matter and grey matter changes in patients with MS indicated that both white matter and grey matter loss occurred early in the disease course, regardless of disease phenotype^50–53^. Evidence also indicates that grey matter damage can occur before white matter atrophy and can occur independently of white matter lesions^54–56^. Results of further longitudinal studies have identified larger decreases in grey matter volumes than in white matter volumes^57–59^ and that grey matter damage is more relevant than white matter injury to clinical outcomes, both concurrent and forthcoming^56,60–62^. Two studies — one in which cortical thickness was estimated^63^ and one meta-analysis of voxel-based morphometry studies^64^ — have revealed statistically significant associations between disability end points and grey matter atrophy^65^, which occurs bilaterally, predominantly in the cingulate, pre-central and/or post-central gyri and the thalami and basal ganglia. Despite these results, global brain volume changes seem to be more strongly associated with clinical outcomes than are regional changes. This observation is unexpected because grey matter loss is thought to underlie disability accumulation. Associations between grey matter volume change and disability accumulation might be masked by the effects of high variability of regional segmentations, which makes clinical application of these regional measures inadvisable at present^62,66^.

### Statements and recommendations

1. We recommend measurement of global brain volume to better gauge global disease burden in patients with MS because brain volume loss is associated with and predicts disability in all clinical MS phenotypes, including the earliest stages of the condition.
2. We recommend measurement of cervical cord area loss because this measure is associated with and predicts disability in all clinical MS phenotypes, including the earliest stages of the condition.
3. Grey matter volume changes in the brain are more pronounced and clinically relevant than white matter volume changes, even in the earliest stages of MS, but their exact relevance in clinical practice is unclear. We recommend further research to clarify this relevance.
4. Some cerebral grey matter regions (including the thalami, basal ganglia and specific cortical areas) are affected particularly strongly by atrophy in MS, but whether the pathological involvement of these areas is relevant in clinical practice remains unclear. We recommend further research to determine the clinical relevance of atrophy in these regions.

## Monitoring therapeutic effect

### Evidence review

#### Brain volume as an outcome measure in randomized clinical trials

Many trials of disease-modifying therapies for MS have included brain atrophy as an outcome measure (Table 1). Most early studies of interferon-β (IFNβ) and glatiramer acetate did not include preplanned brain volume measures as secondary MRI outcomes. Those that did include a sound comparison of brain volume changes between intervention arms or between intervention and placebo arms produced mixed results^67^.

**Table 1:** Brain atrophy outcomes in pivotal trials of approved disease-modifying drugs

Drug	Clinical trial	Phenotype	Comparator	Time frame	Software	Treatment favoured
IFNβ1a (SC)	ETOMS^68^	CIS	Placebo	0–24 months	SIENA^113^	IFNβ1a (SC)
	REFLEX^155^	CIS	Placebo	0–24 months	SIENA^113^	None
	PRISMS^156^	RRMS	Placebo^a^	0–6 years	Kappos et al.^156^	None
IFNβ1b (SC)	BENEFIT^157^	CIS	Placebo^a^	0–36 months	SIENA^113^	None
	EUSPMS^158^	SPMS	Placebo	0–36 months	Losseff et al.^8^	None
	Montalban et al.^76^	PPMS	Placebo	0–24 months	SPM^51^	None
IFNβ1a (IM)	Rudick et al.^69^	RRMS	Placebo	0–24 months	Rudick et al.^70^	None
	Leary et al.^90^	PPMS	Placebo	0–24 months	Fox et al.^140^	None
Glatiramer acetate	PRECISE^159^	CIS	Placebo	0–36 months	SIENA^113^	None
	Sormani et al.^160^	RRMS	Placebo^a^	0–18 months	SIENA^113^	Glatiramer acetate
	PROMISE^75^	PPMS	Placebo	0–36 months	Wolinsky et al.^75^	None
Teriflunomide	TOPIC^161^	CIS	Placebo	0–24 months	Miller et al.^161^	None
	TEMSO^162^	RRMS	Placebo	0–24 months	Wolinsky et al.^163^	None
	TEMSO^164^^,b^	RRMS	Placebo	0–24 months	SIENA^113^	Teriflunomide
Dimethyl fumarate	DEFINE^82^	RRMS	Placebo	6–24 months	SIENA^113^	Dimethyl fumarate
	CONFIRM^165^	RRMS	Placebo	0–24 months	SIENA^113^	None
Natalizumab	AFFIRM^77^	RRMS	Placebo	0–24 months	Rudick et al.^70^	None^d^
	SENTINEL^166^	RRMS	Placebo^c^	0–24 months	Rudick et al.^70^	None^d^
	ASCEND^167^	SPMS	Placebo	24–96 weeks	SIENAX^113^	None
Fingolimod	FREEDOMS 1 (ref.^168^)	RRMS	Placebo	0–24 months	SIENA^113^	Fingolimod
	FREEDOMS 2 (ref.^169^)	RRMS	Placebo	0–24 months	SIENA^113^	Fingolimod
	TRANSFORMS^81^	RRMS	IFNβ1a (IM)	0–12 months	SIENA^113^	Fingolimod
	INFORMS^91^	PPMS	Placebo	0–36/60 months	SIENA^113^	None
Alemtuzumab	CARE-MS 1 (ref.^79^)	RRMS	IFNβ1a (SC)	0–24 months	Rudick et al.^70^	Alemtuzumab
	CARE-MS 2 (ref.^80^)	RRMS	IFNβ1a (SC)	0–24 months	Rudick et al.^70^	Alemtuzumab
Ocrelizumab	OPERA 1 (ref.^170^)	RRMS	IFNβ1a (SC)	24–96 weeks	SIENA^113^	Ocrelizumab
	OPERA 2 (ref.^170^)	RRMS	IFNβ1a (SC)	24–96 weeks	SIENA^113^	Ocrelizumab
	ORATORIO^171^	PPMS	Placebo	24–120 weeks	SIENA^113^	Ocrelizumab
Cladribine	ORACLE^172^	CIS	Placebo	0–24 months	SIENA^113^	None
	CLARITY^83^	RRMS	Placebo	6–24 months	SIENA^113^	Cladribine

CIS, clinically isolated syndrome; IFN, interferon; IM, intramuscular; PPMS, primary progressive multiple sclerosis; RRMS, relapsing–remitting multiple sclerosis; SC, subcutaneous; SIENA, Structural Image Evaluation, using Normalization, of Atrophy; SPMS, secondary progressive multiple sclerosis. ^a^Includes a period receiving the active drug. ^b^Reanalysis of TEMSO trial data using SIENA. ^c^As an add-on to IFNβ1a (IM). ^d^Results favoured natalizumab in the 12–24-month period.

The only study of IFNβ that provided evidence for a positive effect of treatment of brain atrophy was the ETOMS trial^68^. In this study, accrual of atrophy was reduced by 30% in patients with clinically isolated syndromes who received low-dose subcutaneous IFNβ1a compared with patients who received placebo^68^. In several trials — particularly the trial of intramuscular IFNβ1a in RRMS^69,70^ — negative results were at least partly attributed to a pseudoatrophy effect, caused by brain volume loss linked to the presumed treatment-associated resolution of inflammatory activity and oedema. In the RRMS intramuscular IFNβ1a trial, significant differences that favoured treatment with IFNβ1a were only observed in the second year^69,70^. A post-hoc analysis of grey matter and white matter atrophy during the 2 years of the trial confirmed this finding and indicated that pseudoatrophy of white matter contributed most to the observed effect^71^. The same effect has been described in observational studies of patients taking natalizumab^72^ or IFNβ^73^, although more research is needed to confirm these findings. Results with glatiramer acetate were also mixed, though some nonprimary analyses have suggested a positive effect of the treatment in patients who received glatiramer acetate from the beginning of the trial when compared with those who received the treatment later^74^. Trials of IFNβ and of glatiramer acetate in progressive MS have been negative^75^ or have also suggested a pseudoatrophy effect^76^.

Trials of natalizumab provided a clear demonstration of pseudoatrophy. In the AFFIRM trial^77^, brain volume decreases among patients who received natalizumab were larger in the first year than among patients who received placebo, but the observation was reversed in the second year. Subsequent clinical trials of newer drugs (including fingolimod, dimethyl fumarate, teriflunomide, ocrelizumab and alemtuzumab) have all incorporated brain volume measures as secondary or tertiary outcomes, and results have been positive overall^78^, although studies are not readily comparable. Of note, in studies of powerful anti-inflammatory drugs against active comparators, the trial drugs have been superior at decreasing accrual of atrophy^79–81^, indicating that the pseudoatrophy effect can be overcome by the beneficial effects of anti-inflammatory drugs on neurodegeneration in MS. Strategies to minimize the effect of pseudoatrophy on clinical measures include, but are not restricted to, obtaining baseline measurements once the anti-inflammatory effect is well established (for example, re-baseline with MRI at 6 or 12 months after treatment initiation)^82,83^.

Further support for the clinical relevance of brain volume outcomes in trials of treatment for RRMS comes from a meta-analysis that included >13,500 patients from 13 different clinical trials^84^. The conclusion of the analysis was that the effect of a given therapy on changes in brain volume over 2 years is associated with the effect of the drug on disability outcomes and that this association is, at least in part, independent of its anti-inflammatory effect on active MRI lesions^84^. This close association between brain atrophy and disability outcomes in clinical trials has driven the adoption of brain volume change as a primary outcome in phase II trials in cohorts of patients with progressive MS^85,86^.

#### Spinal cord atrophy as an outcome in randomized clinical trials

Despite the relevance of spinal cord atrophy to long-term disability, this measure has scarcely been used as an outcome in clinical trials^87^; when it has been used, the results have been negative. For example, spinal cord atrophy was an outcome measure in an investigator-initiated study of lamotrigine for neuroprotection in secondary progressive MS, but no differences were seen between the treatment and placebo arms^88^. Spinal cord atrophy measures have been used in several other studies in progressive MS^89^ but the atrophy and clinical results have either been negative^76,90^ or were not published with the rest of the trial^91^.

#### Brain volume and spinal cord atrophy to monitor clinical treatment response

The relevance of brain volume measures to the evolution of disability in MS clinical trials is beyond any doubt^84^. The evidence from trials is complemented by that from studies of individual-level data from clinical trials^92,93^ and from observational studies of real-world cohorts^25,94^, which confirm a close association between brain volume changes with therapy and concurrent^95^ or subsequent^96^ disability progression. These studies also indicate that the association between brain volume loss and disability progression is independent of clinical and MRI inflammatory markers.

Most models for the prediction of disability progression have included brain volume change combined with either the appearance of new T2 lesions or the presence of clinical relapses^25,92–94^. Brain volume changes have also been proposed as an addition to the ‘no evidence of disease activity’^97,98^ outcome measure so as to enable assessment of neurodegenerative processes as well as inflammatory processes, with the aim of achieving full remission that includes an absence of disease-specific neurodegeneration; the proposed cut-off for this measure is –0.4% change in volume per year^24^. In a potentially more realistic ‘minimal evidence of disease activity’ approach^99^, a less stringent cut-off has been suggested that would allow for pseudoatrophy-driven brain volume loss^25^. However, all these data need confirmation, and different cut-offs might be needed for different calculation methods and for different drugs or groups of drugs according to different temporal patterns of brain volume effects of each drug^6,78^.

### Statements and recommendations

1. We recommend the use of whole brain atrophy over a minimum period of 12 months as a secondary end point in clinical trials in MS and even as a primary outcome measure in trials in the progressive forms of MS to show the effects of the drug on the neurodegenerative component of the disease.
2. Ongoing and forthcoming trials are expected to include grey matter volume loss as an outcome measure, as atrophy in the grey matter compartment is more substantial and more clinically relevant than atrophy in the white matter and is likely to be affected less by pseudoatrophy; however, data on pseudoatrophy remain discordant and we recommend further research to clarify the contribution of grey matter atrophy.
3. Pseudoatrophy effects mostly occur within the first 6–12 months from treatment initiation with any anti-inflammatory therapy, so we recommend re-baseline MRI at 6–12 months after initiation of any therapy to mitigate the impact of pseudoatrophy on outcome measures.
4. Associations between treatment effects on brain volume and disability have been demonstrated in clinical trials and indicated by evidence at the individual level, but we recommend further research to confirm these associations before brain volume can be considered for use as a treatment-monitoring tool.
5. Use of spinal cord atrophy as a treatment-monitoring tool in clinical trials and in clinical practice has been scarce, but the rate of spinal cord atrophy is faster than that of brain atrophy and methodological advances could improve reproducibility and reliability, so we recommend further research to establish the role of spinal cord atrophy for treatment monitoring.

## Barriers to clinical implementation

### Evidence review: technical barriers

Several technical aspects of image acquisition and quantification can affect the measurement of brain and spinal cord volumes and thereby affect the accuracy of estimated values. These technical barriers are discussed below.

#### Acquisition protocols

The choice of the acquisition parameters (usually repetition time, echo time, inversion time or flip angle) is usually based on the image contrast, as assessed visually by an expert neuroradiologist. Changes in scan parameters, which tend to happen in a clinical environment, affect quantification and hamper reliable cross-sectional and longitudinal comparisons. Image contrast also depends greatly on the age of the population that undergoes MRI. The Alzheimer’s Disease Neuroimaging Initiative^100^ has made a large effort to homogenize acquisition protocols across vendors.

#### Gradient distortion

By design, the gradients applied to the magnetic field in MRI are generally not uniform, which affects the geometry of the image. Small displacements of the patient’s head in the *z* axis have a notable effect on the estimated brain volume change^101^. Positioning of the patient identically across scanning sessions can minimize this effect, but this is time-consuming and difficult; a better solution is to apply approaches developed by MRI scanner manufacturers for 3D correction for the gradient nonlinearity effect^102^.

#### Intrascanner variability

Any MRI-derived measure is inherently variable, even when technical and physiological conditions are controlled^103–108^. Global estimates, such as that of the whole brain volume, are the least variable (<1%)^106^, whereas measures of smaller structures, such as the amygdala, are much more variable (~5%)^104,105^. Such variability must be taken into account because changes that are smaller than the estimated variability cannot be reliably detected. This limitation is highly relevant to small grey matter structures and when follow-up periods are short because the expected change is small^23^.

#### Movement

Movement of the patient during image acquisition generates characteristic artefacts that affect image quality; as a result, estimated volumes are substantially decreased^109^. Visual verification of image quality is important because the problem is resolved when the only images included in an analysis are those that an expert considers artefact free^109^. Various approaches have been developed to correct for movement, but an accurate method is still not available^110^.

#### Scanner system upgrades and interscanner variability

Scanner upgrades are unavoidable, particularly during the course of longitudinal studies, and can affect the image contrast even if the same acquisition parameters are used. Previous studies have shown that the system upgrade should be included as a variable in the statistical analysis^103,111,112^. Quantification methods based on the subtraction of images, rather than on differences in brain parenchymal fraction between two time points, seem to be more sensitive to system upgrades^113^, although no studies have been performed to confirm this observation. Reliable quantification of longitudinal changes in MS requires scans to be acquired with the same magnet and exactly the same sequence protocol. Variability between different scanners is higher than all the factors above together^108^. If data acquired in different scanners need to be merged, a variable that accounts for the scanner should be taken into consideration.

### Evidence review: confounding factors

Numerous factors can have confounding effects on the quantification of brain volume (and its changes) and thereby cause overestimation or underestimation^114^. These factors are discussed below.

#### Age, sex and brain size

Several physiological factors influence brain volume estimations in healthy individuals. Studies of healthy elderly individuals have demonstrated ongoing brain volume loss, which tends to accelerate with age^115^. This age-related effect is particularly pronounced for specific CNS structures, such as the hippocampus^116^.

Sex is another key factor in brain volume changes. Sex differences in global brain size in humans are well established; on average, the total volume of men’s brains is ~10% larger than that of women’s brains^117^. Differential patterns of age-related brain volume loss^118^ and sex-specific differences in brain morphology have also been demonstrated^119,120^. Global and regional volumetric studies have suggested that hormonal status can contribute to these sex-related differences^121^.

#### Diurnal fluctuations and hydration state

Studies of healthy individuals have shown that estimations of brain volume fluctuate with the time of scanning and the hydration state of the individuals. Analysis of MRI data from patients with MS (*n* = 755, 3,269 scans) and from participants in the Alzheimer’s Disease Neuroimaging Initiative (*n* = 834, 6,114 scans) revealed that time of day had a notable effect on estimates of the brain parenchymal fraction in both groups. Brain volumes were substantially larger in the morning^122^, and the effect size was comparable to the yearly rate of brain atrophy in MS and in healthy elderly people^122^. Similarly, in studies in which hydration status was manipulated by overnight thirsting and subsequent drinking of water, hydration-related changes in brain volume were as large as –0.55% on dehydration and +0.72% on rehydration^123^.

#### Lifestyle and risk factors

Many lifestyle factors, including physical activity^124^, influence estimates of brain volume. A higher level of alcohol intake has been associated with a higher rate of brain atrophy over a 6-year period^115^ and with a specific pattern of regional involvement of the white matter and grey matter^125^. A similar effect has been described for cigarette smoking and substance abuse (for example, marijuana use)^115,126^. Many systemic conditions, such as diabetes, chronic kidney disease, hypertension, obesity and vascular conditions can also accelerate brain atrophy^115,127,128^.

#### The MS brain

All confounding factors previously discussed can interact with features of MS and affect estimates of brain atrophy in patients with the disease; these interactions can also affect comparisons between groups. For instance, more severe brain atrophy has been observed in patients with MS who have one or more cardiovascular risk factors^129^, although their impact on longitudinal assessments might be limited, as vascular risk factors were not associated with greater brain volume loss during 3.5 years of follow-up in the same study^129^. In addition, white matter lesions in MS influence the accuracy of most available software for estimation of atrophy because they alter the image intensity histogram and influence the detection of intensity borders between grey matter, white matter and cerebrospinal fluid (CSF). This effect can be minimized by use of lesion filling techniques^130,131^, which enable replacement of lesions in the image with voxels that have intensities that closely resemble normal-appearing white matter.

#### Pseudoatrophy

As discussed above, studies of the correlation between inflammatory disease activity (new T2 and/or gadolinium enhancing lesions) and brain volume have shown that inflammation can cause a transient increase in brain volume. This increase can dramatically resolve following treatment with steroids^132^ or other disease-modifying drugs, and the resultant reduction in brain volume can be erroneously interpreted as atrophy^133^.

### Evidence review: volumetry tools

Several free-to-use online libraries of software for neuroimaging analyses include fully automated pipelines for quantification of brain volume (Table 2). On the basis of the current literature that relates to this software, these software tools can be classified into two broad categories. The first are ‘segmentation-based’ tools, which use a priori localization-related and intensity information to classify the brain voxels of each MRI without using information from brain MRI images taken at different time points. These tools do not enable direct evaluation of volumetric changes over time. This type of software is mostly used in cross-sectional analyses. The second are the ‘registration-based’ tools, which enable comparison of brain MRI images from the same individual acquired over time and are based on an initial registration step; this type of software is used in longitudinal analyses^134^.

**Table 2:** Available brain and spinal cord volumetry tools

Tool	Freely available?	Measures	Major limitations^a^
SIENAX	Yes	Global and regional brain volumes for cross-sectional comparisons	Segmentations are affected by the presence of brain lesions
SPM/VBM	Yes^b^	Global and regional brain volumes, pixel-to-pixel statistical comparisons between two groups or time points	Segmentations are affected by the presence of brain lesions
GIF	Yes	Regional brain volumes for cross-sectional comparisons	Time consuming; data analysed remotely
Atropos	Yes	Regional brain volumes for cross-sectional and longitudinal comparisons	Limited information about the method as it has not been used extensively
FreeSurfer	Yes	Cortical thickness, global and regional grey matter and white matter volumes	Time-consuming; requires manual correction of the segmented surfaces; segmentations are affected by the presence of lesions
CIVET	No	Cortical thickness	Software not freely available
SIENA	Yes	Percentage brain volume change between two time points	Only provides global measures that include grey matter and white matter
SIENA-XL	No	Grey matter and white matter volumes for longitudinal comparisons	Software not freely available
SIENAX-MTP	No	Grey matter and white matter volumes for longitudinal comparisons	Software not freely available
BBSI	Yes	Percentage brain volume change between two time points	Only provides global measures that include grey matter and white matter
CLADA	No	Cortical thickness	Software not freely available
NeuroQuant (FDA clearance and CE mark received)	No	Global and regional grey matter volumes	Validation of results is only external; segmentations affected by the presence of brain lesions; only images from the scanner can be analysed (that is, filled images cannot be used)
Icometrix (FDA clearance and CE mark received)	No	Global and regional grey matter volumes	Whole verification of the results is not direct; data analysed remotely
Biometrica (CE mark received)	No	Global and regional grey matter volumes	Whole verification of the results is not direct
Quantib (FDA clearance and CE mark received)	No	Global and regional grey matter volumes	Whole verification of the results is not direct
Cordial	Yes	Spinal cord volume	Limited information about the method as it has not been used extensively
Spinal Cord Toolbox	Yes	Spinal cord area, volume and length	Regions of interest should be edited and manually corrected
JIM	No	Spinal cord area, volume and length	Needs several reference marks for accurate estimates

BBSI, Brain boundary shift integral; CE, Conformité Européenne; CLADA, cortical longitudinal atrophy detection; GIF, Geodesical information flows; JIM, Jacobian integration method; SIENA, Structural Image Evaluation, using Normalization, of Atrophy; SPM, statistical parametric mapping; VBM, voxel-based morphometry. ^a^Not exhaustive, only major limitations are included. ^b^SPM itself is free but a MATLAB licence is needed.

Most segmentation-based software packages provide measures of total brain volume, grey matter volume and white matter volume based on the partial volume estimation (PVE) of each tissue in each voxel. The initial step is assignment of the PVE to a given brain voxel on the basis of its intensity and the intensities of the surrounding voxels^113^. To improve the segmentation, the a priori spatial information for each voxel can be included, thereby increasing the probability that each voxel belongs to specific tissue type on the basis of its location^135^, although the accuracy of this step strongly depends on the anatomical similarity between the MRI image and the a priori tissue maps used. To avoid problems due to an anatomical mismatch with the atlas, only MRI images with high anatomical similarity should be used to provide the voxel location information^136^. Use of different anatomical maps, such as probability maps of tissues or structure labelling maps, can also offer improvements^137^. Other approaches that do not depend on the PVE can provide a measure of cortical thickness by calculating the distances between pairs of voxels at the grey matter–white matter and grey matter–CSF interfaces perpendicular to the grey matter–white matter surface interface. These methods tend to be more susceptible than some of the previously mentioned methods to the low-intensity contrast between tissues because they heavily rely on the gradient intensities between tissue interfaces^138,139^.

Registration-based software packages provide measures of total brain volume, grey matter volume and white matter volume changes by comparison of serially acquired MRI images from the same individual. A common preliminary step in most of these procedures is registration of all MRI images from the same subject on the same virtual space. The first such software packages that were used in longitudinal analyses^113,140^ involved registration of two MRI images of the same individual and measurement of whole brain volume change by analysing the shift of the parenchyma–CSF border over time. Newer approaches apply different methods to enable assessment of grey matter and white matter volume changes. In one, for each voxel, the intensity information from neighbouring voxels at each time point is used^141^. In another, a new intensity harmonization scheme is applied to all MRI images from one individual, with the aim of assigning similar intensity to voxels with similar content of PVE^142^. Another approach, known as the Jacobian integration method^143,144^, is based on local assessment of relative volumetric differences between two MRI images of the same individual, one of which is usually the baseline image; the net sum of all local volumetric changes provides an estimate of total volume changes over time. Finally, cortical thickness changes can be detected by the use of a within-subject template (an MRI image created by merging all MRI images from one individual) to improve cortical thickness estimation at each time point, or by fitting a subject-specific cortical deformable model at each time point^145,146^.

Assessment of spinal cord atrophy is more difficult than brain segmentation owing to particular anatomical (higher mobility and smaller dimensions than the brain) and imaging (lower tissue contrast) features of the spinal cord. Semiautomated (Cordial)^147^ and automated (Spinal Cord Toolbox)^148^ tools have now been developed, based on deformable models. These promising new software tools still need to be extensively validated on independent datasets before they can even be considered for use in clinical practice.

Academic software packages have important advantages over commercial software packages, such as the fact that they have been validated in many studies under a plethora of different MRI conditions over the past decade. However, they have the severe limitation of being highly technically demanding and their use is therefore limited to centres that are specialized in MRI processing. In addition, clinical application of software to support diagnosis or care is only permitted with products that have received the “Conformité Européenne” (CE) mark in Europe or FDA clearance in the USA. For this reason, translation of imaging analysis software tools to clinical practice is challenging and almost unfeasible for academic neuroimaging laboratories.

In the past 10 years, several companies have proposed centralized MRI reading services, often using their in-house software for quantification of atrophy (Table 2). Four software packages have been approved for use in Europe and three of these have also received FDA clearance in the USA. The IcoBrain MS (Icometrix, previously MSmetrix)^149^ quantifies cross-sectional volumes with software based on Nifty Seg and quantifies longitudinal changes in grey matter and white matter with software that implements Jacobian integration. NeuroQuant (CorTechs Labs)^150^ provides both cross-sectional and longitudinal quantification of atrophy^151^, building on approaches already developed by previous methods^138^. Biometrica MS (Jung Diagnostics) builds on developments of Statistical Parametric Mapping, a software library for neuroimaging analysis, for atrophy measurement and of Lesion Segmentation Tool software for automatic lesion segmentation^152,153^. Quantib Brain (Quantib) is a platform that is integrated into the General Electric MRI scanner and can assess cross-sectional brain volumes and longitudinal changes in volume. IcoBrain MS and Biometrica MS are offered as remote analysis services, Quantib Brain can be run locally or on a vendor console (General Electric), and NeuroQuant can be a remote analysis service or local installation. All packages have the CE mark and, with the exception of Biometrica, FDA clearance. These certifications guarantee standardization of procedures and results, meaning the software can be used as medical devices.

Importantly, the companies must provide the magnitude of the error in their results, and health care professionals should use this information to validate or discard findings of analyses. All four commercial software packages have been evaluated scientifically to some extent but not completely. To our knowledge, only MSmetrix has been validated by an independent group in the context of MS^154^. Furthermore, the real-world clinical value of these software packages has not yet been assessed, and the procedures are not widely reimbursed (with a few exceptions, such as in the USA). Although promising, these analytical approaches should therefore be more extensively validated by expert groups in the field of MRI preprocessing, especially in the context of MS^134^, before they can be considered for use in the routine clinical setting.

### Statements and recommendations

1. We recommend appropriate management of several scanner-related factors (including, but not limited to, variation in acquisition protocols, different scanner systems and upgrades, movement artefacts and gradient distortions) to ensure reliability of brain volume estimates, particularly at an individual patient level.
2. We recommend appropriate management of physiological and MS-related factors (including, but not limited to, age, sex, hydration status, time of day, steroid use and MS-related parenchymal alterations).
3. Brain volume measures are software-dependent so the use of software that has been approved as a medical device and independently evaluated in MS is a prerequisite; we recommend further research to validate existing software tools in MS and assess their clinical value.

## Conclusions and future directions

Based on the evidence reviewed, the idea that brain volume changes and, to a lesser extent, spinal cord atrophy are helpful predictors of the evolution of MS before initiation of therapy is undisputed, so these measures could be valid treatment-decision tools. The evidence reviewed also supports the idea that brain volume measures have value in monitoring the effects of MS drugs as part of the no evidence of disease activity outcome measure or minimal evidence of disease activity outcome measure. However, several potential sources of substantial error remain, including, but not limited to, differential effects of drugs on brain volume measures, confounding physiological and technical factors and the performance and value of volumetric tools. To make implementation of volume measurements in clinical practice feasible, these potential sources of error need to be accounted for and appropriately managed, and further research is needed to ensure the accuracy and reliability of the measurements.

## Glossary

Brain parenchymal fraction: The percentage of the intracranial volume that is occupied by brain parenchyma, calculated as the total intracranial volume minus the volume of cerebrospinal fluid divided by the total intracranial volume.
Partial volume estimation: (PVE). The proportions of white matter, grey matter and cerebrospinal fluid present in each voxel of an MRI image.
Deformable model: A mathematical construct capable of representing a broad range of shapes that can be used in MRI image registration or segmentation.
Nifty Seg: An open-source image analysis tool developed at University College London to perform segmentations of MRI images.
